# A promising drug delivery candidate (CS-g-PMDA-CYS-fused gold nanoparticles) for inhibition of multidrug-resistant uropathogenic *Serratia marcescens*

**DOI:** 10.1080/10717544.2020.1809557

**Published:** 2020-09-04

**Authors:** Ping Shi, Rajendran Amarnath Praphakar, Sadhasivan Deepa, Kannan Suganya, Prashant Gupta, Riaz Ullah, Ahmed Bari, Marudhamuthu Murugan, Mariappan Rajan

**Affiliations:** a Management Office of Drug Clinical Trial Research, Affiliated Hospital of Qingdao University, Qingdao, Shandong; b Biomaterials in Medicinal Chemistry Laboratory, Department of Natural Products Chemistry, School of Chemistry, Madurai Kamaraj University, Madurai, India; c Department of Microbial Technology, School of Biological Sciences, Madurai Kamaraj University, Madurai, India; d Department of Balroga, Govt. Ayurved College, Raipur, Chhattisgarh, India; e Department of Pharmacognosy, College of Pharmacy, King Saud University, Riyadh, Saudi Arabia; f Department of Pharmaceutical Chemistry, College of Pharmacy, King Saud University, Riyadh, Saudi Arabia

**Keywords:** Antibiotic resistance, gold nanoparticle, pH-sensitive drug delivery, *Serratia marcescens*

## Abstract

Antibiotic resistance amongst microbial pathogens is a mounting serious issue in researchers and physicians. Various alternatives to overcome the multidrug-resistant bacterial infections are under search, and biofilm growth inhibition is one of them. In this investigation, a polymeric drug delivery system loaded with multi-serratial drugs to improve the delivery of drugs against urinary tract infection causative *Serratia marcescens*. The chitosan grafted pyromellitic dianhydride – cysteine (CS-g-PMDA-CYS) was conjugated with AuNPs by using the –SH group of CYS and RF (rifampicin) and INH (isoniazid) were loaded in AuNPs-fused CS-g-PMDA-CYS system. Several physicochemical techniques characterized this fabricated AuNPs/RF/INH/CS-g-PMDA-CYS system. The successful encapsulation of RF and INH in AuNPs-fused CS-g-PMDA-CYS polymer had confirmed, and it observed the loading capacity for RF and INH was 9.02% and 13.12%, respectively. The *in vitro* drug discharge pattern was perceived high in pH 5.5 compared with pH 7.4. The AuNPs/RF/INH/CS-g-PMDA-CYS escalates 74% of *Caenorhabditis elegans* survival during *Serratia marcescens* infection by aiming biofilm development and virulence in *S. marcescens*. Author postulate that the fabricated system is a promising drug carrier and delivery system for inhibition of multidrug-resistant bacterias like *S. marcescens*.

## 1. Introduction

Antibiotics are chemical drugs used to cure for the infections of bacteria, and antibiotic resistance (AR) happens when bacteria alter the reaction to the usage of antibiotics. AR is affecting irrespective of gender, age, or geography and mounting as one of the major threats to world health, food safety, and progress (https://www.who.int/news-room/fact-sheets/detail/antibiotic-resistance). A high quantity of bacterias diseases exhibits antibiotic resistance. *Serratia marcescens* is a leading resourceful urinary tract, and nosocomial pathogen complicates to exterminate due to biofilm growth mode fully. Flagellum-mediated motility, such as swimming and swarming, is also crucially important. Biofilms inhabitants necessarily require a higher (thousand times) concentration of antibiotics for the eradication. Maximum *S. marcescens* strains are resistant to routine antibiotics, hence, hard-tackling issues for public health. The majority of the bacterial resistance mechanisms to antibiotics are extraneous to nanoparticles (NPs). NPs are directed to the cell wall without penetrating the cell, which strengthens that NPs would be unlikely prone to promote bacterial resistance than antibiotics. Thus, novel and exciting NPs-based antimicrobial materials activity is the focus of attention (Labuschagne et al., 2014; Amarnath Praphakar et al., 2018; Mehnath et al., 2019).

The aim of this study to develop cost-effective and safe drug delivery systems, which can significantly increase the site-specific activity, compatibility, bioavailability, and high therapeutic index of the corresponding drugs (Rajan & Raj, 2013). The utilization of various nanoscale carrier materials, including liposomes, polymers, metal nanoparticles, and micelles in antibacterial-drug carrier systems, keeps on being a subject of extraordinary intrigue (Zhu et al., 2011; Hu et al., 2012; Rajan & Raj, 2012; Yuan et al., 2019). Among these various nano-sized drug delivery systems, polymeric materials primarily aim to fabricate a drug delivery system with excellent biocompatibility and less toxicity (Chaubey & Mishra, 2014).

Chitosan (CS) is a natural cationic polymer of 2-amino-2-deoxy-β-d-glucan connected through glycosidic bonds (Lai & Shum, 2015). The superb biocompatibility, biodegradability, and low immunogenicity of CS make it helpful in pharmaceutical applications as a vehicle for bioactive agents, DNA, and proteins, and so forth (Sunil et al., 2004; Rajesh et al., 2012; Pengpong et al., 2014). CS arrives at cell films all the more effectively and permits ionic linkage with anions on the cell layer surface because of its cationic property (Luo et al., 2005). Besides, the mucoadhesive feature likewise expands its maintenance over-focused substrates. Be that as it may, CS gives a few restrictions, unsteadiness, and ensnarement proficiency of loaded drugs (Amarnath Praphakar et al., 2017). Expanded examinations gave an account of CS hydrophilic/hydrophobic adjustments in an offer to defeat its downsides in sedate conveyance frameworks (Amarnath Praphakar et al., 2018, 2019).

The number of metal nanoparticles is under research in biomedical applications, especially the gold nanoparticles (AuNPs). Traditionally, gold nanoparticles are the most ancient illustration of nanotechnology, which demonstrates nanoparticle size in its complete form, and these metal nanoparticles considered as potential drug delivery vehicles and bio-availability enhancers of co-administered drug (Anand & Neetu, 2010; Singh et al., 2016). AuNPs have been applied in several therapeutic areas, like gene delivery, radiotherapy boost, thermal ablation, DNA-binding agents, highly sensitive diagnostic assays, and biomolecular sensing (Huang et al., 2007; Yang & Cui, 2008). Considerable surface-to-volume proportion and quick functionalization through thiol bonds render AuNP appealing for multiple applications in the biomedical field (Brown et al., 2010).

Research has been done to associate polymers with metal nanoparticles in an attempt to prepare a hybrid inorganic metal/polymer nanocomposite (Camargo et al., 2009). The prospective arrangement of the benefits of inorganic metals (e.g. high hardness, high warm strength, top refractive list, and stability) with those of polymers (e.g. processability, adaptability, and weightless) can empower a broad scope of utilization for these nanocomposites (Mohammed Adnan et al., 2018). Here, an inorganic metal/polymer contains a hybrid nanocomposite was prepared for the successful delivery of antibacterial drugs against nosocomial *Serratia marcescens*. The nanocomposites regarding the chemical modifications, loading capacity, surface morphology, *in vitro* drug release profile, biological investigation, and therapeutic potential. Successfully CS and AuNPs-based hybrid inorganic metal/polymer nanocomposite was prepared for the first time for the delivering dual antimicrobial drugs (RF and INH) against nosocomial *S. marcescens*.

## 2. Materials and methods

### 2.1. Materials

Gold chloride trihydrate (HAuCl_4_. 3H_2_O) and sodium citrate tribasic dihydrate were purchased from Sigma Aldrich, India. Rifampicin (RF) and isoniazid (INH) were purchased from SRL, India. Chitosan (CS), pyromellitic dianhydride (PMDA), l-cysteine (CYS), NaOH, and HCl were received from Himedia, Mumbai, India. Other chemicals and solvents were used in delivered form without any additional refinement. The double-distilled (DD) water used for the reactions and the cleaning processes.

### 2.2. Synthesis of gold nanoparticles (AuNPs)

The average diameter of ∼ 13 nm of gold nanoparticles was prepared from HAuCl4 by the citrate reduction method (Aili et al., 2008). Concisely, 1 mM HAuCl4 was liquefied in 50 mL DD water and stirred under reflux condition. Then, the rapid addition of 38.8 mM trisodium citrate in 5 mL water into the HAuCl4 solution and stirred for 15 min results in a color change from pale yellow to wine red. The suspension was stirred without heating for 15 min to reach the room temperature.

### 2.3. Synthesis of chitosan-graft-pyromellitic dianhydride-cysteine (CS-g-PMDA-CYS)

The grafted polymer of chitosan with pyromellitic dianhydride and cysteine was synthesized through the procedure of the previous report (Zhao et al., 2017). Typically, CYS (0.5 g) and CS (1 g) were dissolved in 20 mL 1% acetic acid and diluted with methanol. Subsequently, the suspended solution of PMDA (6 g) in 6 mL methanol was poured dropwise into the above solution. The resultant combination was mixed vigorously in a magnetic stirrer for 24 h at room temperature. The obtained grafted gel was filtered and mixed with ethanol under stirring for an additional 5 h. Then, the solution was washed with 0.1 M NaOH, deionized water, and 0.1 M HCl to remove the unreacted PMDA and CYS. Finally, the gel ‘CS-g-PMDA-CYS’ was dried and lyophilized under vacuum at -40oC for 48 h and stored in vacuo. The obtained CS-g-PMDA-CYS product yield found to be 82.67%.

### 2.4. Synthesis of RF and INH loaded CS-g-PMDA-CYS conjugated AuNPs (AuNPs/RF/INH/CS-g-PMDA-CYS)

The synthesis of multi drugs loaded CS-g-PMDA-CYS conjugated AuNPs was carried out by the following reported protocol (Amarnath Praphakar et al., 2017). In this preparation, CS-g-PMDA-CYS (1000 mg) and INH (100 mg) were dispersed in DD water (10 mL). Then, the 5 mL of organic solutions of dichloromethane containing 100 mg RF and span 20 was added dropwise to the above aqueous solution and stirred to the formation of the emulsion as oil in water (O/W). After the complete evaporation of dichloromethane, 0.5 g of AuNPs dispersed in 5 mL distilled water was introduced dropwise to form RF and INH-loaded CS-g-PMDA-CYS-conjugated AuNPs. The mixed solution was sonicated for 20 min to obtain a gel conjugated AuNPs loaded with multi drugs. The resultant product was filtered and lyophilized for 24 h at −40 °C.

### 2.5. FT-IR (Fourier transform infrared spectroscopy)

The functional group and functional modification of samples were examined by using FT-IR (PerkinElmer) with the spectral range 500–3500 cm^−1^ by accumulating 32 scans.

### 2.6. SEM (scanning electron microscopy)

Samples were coated on a glass plate and fixed on the holder by using double adhesive carbon tape, and gold sputtering was applied. Scanning electron microscopy (VEGA3, TESCAN, Czech Republic) was used to examine the surface morphology at 5 kV accelerating voltage. The elemental distribution in the samples was investigated by energy-dispersive X-ray (EDX) analysis.

### 2.7. Transmission electron microscope (TEM)

TEM images of samples were acquired with a transmission electron microscope (FEI Tecnai G2 F20, Eindhoven, Netherlands) at 200 kV accelerating voltage.

### 2.8. Swelling ratio

The sample was placed in test tubes containing various pH buffer solutions (pHs ∼ 5.5 and 7.4) at 25 °C. At predetermined time intervals, the sample was removed from the pH solution and dried by using filter paper, weighed, and further dissolved in the buffer solution. The swelling ratio was determined by utilization of the following equation (Mehnath et al., 2017):

$$\text{Swelling ratio}(\%)=\frac{\text{Swollen weight of the sample}}{\text{Dry weight of the sample}}\times 100$$

### 2.9. Loading capacity (LC) and in vitro drug release profile

The LC of AuNPs/RF/INH/CS-g-PMDA-CYS was measured by dissolving 10 mg of AuNPs/RF/INH/CS-g-PMDA-CYS in 50 mL of pH ∼ 7.4phosphate-buffered saline solution (PBS) comprising ethanol (5% v/v) under vigorous agitation for 24 h. The free amount of RF and INH was measured from the supernatant solution at different time intervals at a wavelength of 473 and 269 nm, respectively, by UV–Vis absorption spectroscopy (Shimadzu 1800, Kyoto, Japan). The LC was determined by utilization of the following equation (Amarnath Praphakar et al., 2019):

$$(\%)\text{LC}=\frac{\text{Total drug amount}-\text{Free drug amount}}{\text{Dry sample weight}}\times 100$$

For the *in vitro* drug release assay, 30 mg of AuNPs/RF/INH/CS-g-PMDA-CYS was immersed in two different phosphate-buffered salines (PBS, 10 mL, pHs ∼ 7.4 and 5.5) and constantly agitated (80 rpm) at 37 °C. At various time intervals, 1 mL of the release medium was withdrawn and analyzed by using UV–Vis absorption spectroscopy (Shimadzu 1800, Kyoto, Japan) at a wavelength of 473 and 269 nm for RF and INH, respectively. A fresh PBS medium replaced the withdrawn medium. The increasing release pattern was determined by utilization of the following equation (Gowri et al., 2018):

$$\text{Cumulative drug release}(\%)=\frac{\text{Amount of drug released}}{\text{Total amount of drug}}\times 100$$

### 2.10. Anti-pathogenic potential against S. marcescens

#### 2.10.1. Minimum inhibitory concentrations (MIC) determination

MIC was determined using the broth microdilution assay, as mentioned in Gowri et al. (2018).

#### 2.10.2. Inhibition of swarming motility

For the swarming motility, 5 µL of *S. marcescens* was dot inoculated in the middle of the swarming agar medium with and without samples. Further, the swarming plates were hatched vertical position for 18 h at 28 °C to observe the swarm pattern.

#### 2.10.3. Inhibition of Prodigiosin pigmentation

About 1% of 1 × 106 CFU ml^−1^ cell suspension of *S. marcescens* was inoculated into BHI medium with the presence and absence of NPs and incubated up to late stationary phase at 28 °C for 18 h. The cells were collected by centrifugation, and the cell pellet was separated with 1 ml of acidic ethanolic solution (4% 1 M HCl in ethanol). The OD of removed Prodigiosin was estimated at 534 nm.

#### 2.10.4. In situ microscopic observation of biofilm

##### 2.10.4.1. Crystal violet binding and light microscopy analysis

In brief, the MTP wells containing 2 mL of fresh BHI broth with a cover glass (1 cm^2^) inoculated with 1% overnight culture of *S. marcescens* with or without nanoparticles and incubated for 16 h. The planktonic cells were detached after incubation by washing the cover glass with DD water. Cells attachment to glass were marked with a watery 0.4% CV stain and observed under a light microscope.

##### 2.10.4.2. High content screening (HCS) analysis of biofilm

The cells of *S. marcescens* were brooded with prepared samples to contemplate the impact on biofilm passing with the assistance of high content imaging system ((HCS) Operetta, Perkin Elmer, Waltham, MA). The cells were vaccinated in 200 μL of sterile BHI for 24 h at 37 °C to frame biofilms in high substance screening plates (Vision Plate™384 wells dark sterile, plate-dark Perkin Elmer, Waltham, MA). The extra medium was expelled after the brooding time frame, and the planktonic cells were evacuated by washing twice with sterile phosphate cradle (50 mM, pH 7.0) to evacuate the biofilms. The wells were brooded with and without the samples for 2 h. Later the film was recolored with live/dead BacLight Bacterial Viability Kit (Invitrogen, Carlsbad, CA) for the live-dead examination. The pictures were taken, and the Z-stack examination was finished with the Harmony programing (Perkin Elmer, Waltham, MA).

### 2.11. Valuation of in vivo therapeutic potential of the drug on C. elegans

#### 2.11.1. Growth and culture conditions of C. elegans and food source

Food sources *E. coli* OP50 of *C. elegans* N2 (wild-type) were obtained from the *C. elegans* Genetics Center. Completely strains were cultured on nematode growth media (NGM) dishes at 15 °C. During pathogenicity assays, *S. marcescens* was cultured in NGM medium and used as a food source instead of *Escherichia coli* OP50.

#### 2.11.2. Influence of drug on the existence of *C. elegans* upon *S. marcescens* infection

To assess the drug and drug combinations on the endurance of *C. elegans* during *S. marcescens* contamination age-synchronized L4 stage wild-type N2 Bristol *C. elegans* creatures (*N* = 20) were moved to a 24-well plate containing sterile M9 cradle enlarged with or without tranquilize mixes. Each very much was seeded with a 3-h developed culture S. *marcescens* and brooded at 20 °C. The worms took care of with *S. marcescens* alone and *E. coli* OP50 alone with no medication was considered as positive and negative controls, separately, and the endurance rate was recorded dependent on pharyngeal siphoning.

#### 2.11.3. Microscopic observation of in vivo colonization

For microscopic examination of in vivo colonization and adherence, *C. elegans* were showing to *S. marcescens* with and without drug combination for 24 h. Ten untreated control and treated nematodes were rinsed with M9 buffer systematically. Further, the motile worms were positioned on a microscopic slide and anesthetized with 1 mM Sodium azide and imagined under a bright field microscope.

### 2.12. Statistical analysis

Statistical reports are given as standard error of the mean (SEM) (*n* = 3). Student’s *t*-test and one-way analysis of variance (ANOVA) were used to determine significant differences between controls and experiments, and between groups. Significant probability is shown as *p ≤ .05 and **p ≤ .01.

## 3. Results and discussion

### 3.1. Synthesis of AuNPs/RF/INH/CS-g-PMDA-CYS

**Figure 1.**
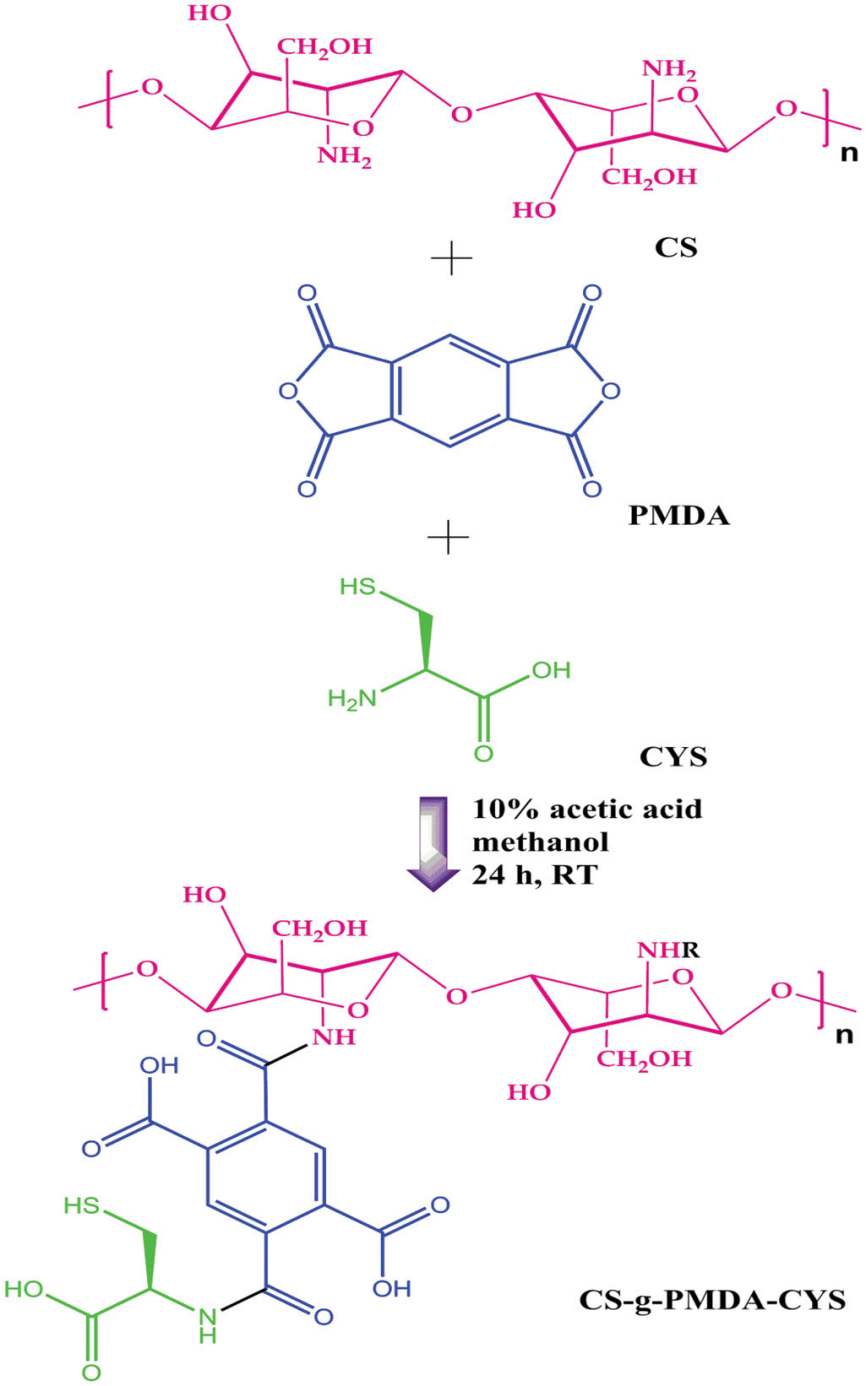
Synthesis of CS-g-PMDA-CYS.

**Figure 2.**
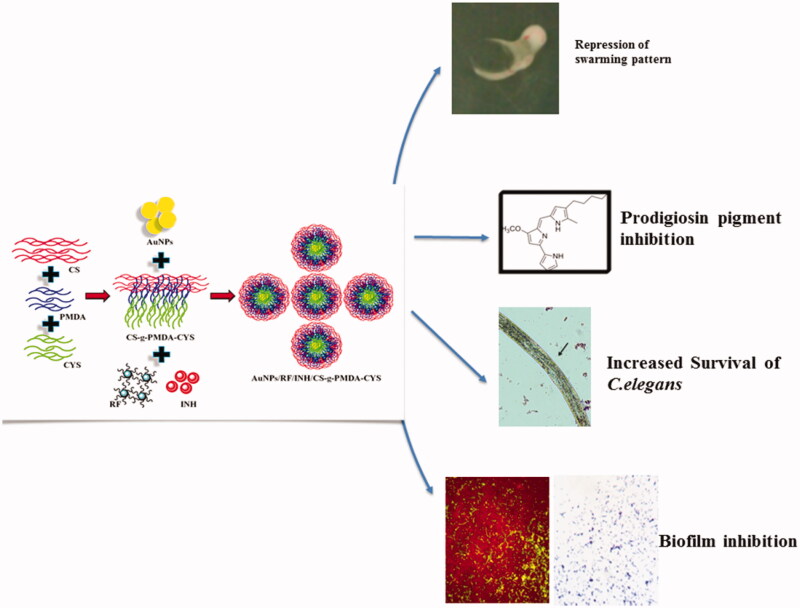
Schematic and sequential representation of the experimental formation of RF and INH-loaded CS-g-PMDA-CYS conjugated AuNPs and their biological outcomes.

In this achievement, we worked to design a self-fluorescent drug delivery system with loaded anti-tubercular drugs RF and INH. AuNPs were synthesized first, following PMDA as a cross-linking agent, which cross-linked CYS and CS results in the formation of the grafted polymer CS-g-PMDA-CYS shown in Figure 1. Hydrophobic RF and hydrophilic INH were encapsulated in CS-g-PMDA-CYS-conjugated AuNPs. The –SH functional group of CYS is conjugated with AuNPs, and the drugs were incorporated in-between the grafted polymer cavities. For the maximum encapsulation, the hydrophobic RF was initially covered with a surfactant span 20 and encapsulated within the polymeric core. The schematic and sequential experimental formation of RF and INH loaded CS-g-PMDA-CYS conjugated AuNPs is given in Figure 2.

### 3.2. FT-IR analyses

**Figure 3.**
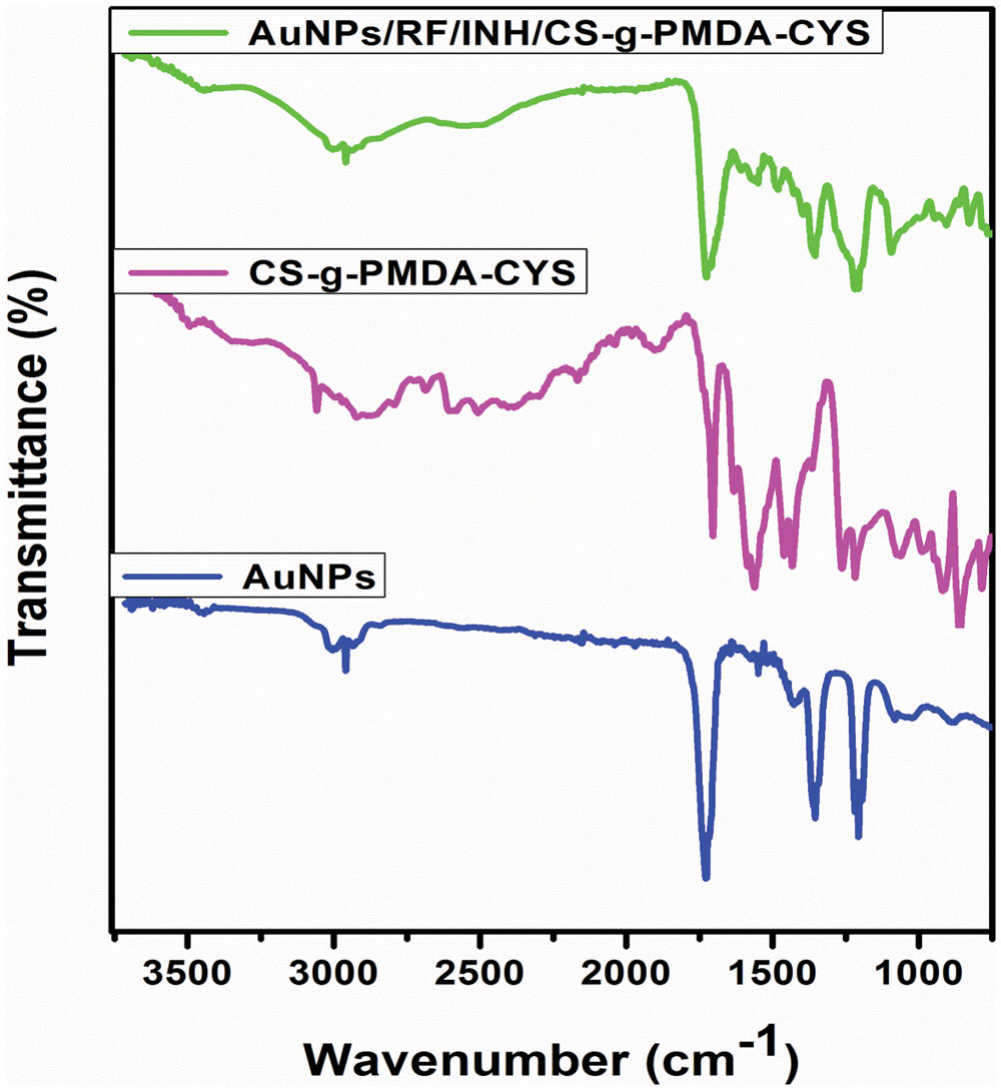
FT-IR images of AuNPs, CS-g-PMDA-CYS, and AuNPs/RF/INH/CS-g-PMDA-CYS NPs.

FT-IR confirmed the structural modification of grafted polymer and the presence of drugs and AuNPs in the polymeric core. According to Son et al., pure RF characteristic absorption peaks appeared at 3300 (–OH), 2903 (–CH_3_), 1735 (C = O), (C–O–C) 1250 cm^−1^ and 896 cm^−1^ (Slowing et al., 2009; Son & McConville, 2011). While Amarnath Prabhakar et al. proved that distinct absorption peaks for pure INH appear at 1665 (amide I), 1551 (amide II), and several peaks in the assortment of 1405–670 cm^−1^ (Amarnath Praphakar et al., 2017). The FTIR spectra of AuNPs in Figure 3(A) shows representative peaks at 2954, 1729, 1547 (COO anti-symmetric stretching), 1359 (COO symmetric stretching), and 1209 reflects the presence of protecting agent citrate. Peaks for CS-g-PMDA-CYS appeared at 3320 (–OH), 2927 (–CH_3_), 2555 (–SH), 1707 (–COOH), 1635 (amide C = O), 1458 (C = C), and 1250 cm^−1^ (C–O–C) (Figure 3(B)). AuNPs/RF/INH/CS-g-PMDA-CYS shows absorption bands at 3218 (–OH), 2919 (–CH_3_), 1729 (C = O), 1707 (–COOH), 1618 (amide C = O), 1547, 1481, 1256, and 897 representing the presence of RF, INH, AuNPs, and CS-g-PMDA-CYS polymer (Figure 3(C)). The peak at 2555 of CS-g-PMDA-CYS was disappeared in the final spectrum, strongly suggesting that the –SH functional group of CS-g-PMDA-CYS was conjugated onto the AuNPs. It suggests that the CS-g-PMDA-CYS polymer was conjugated vigorously on AuNPs with RF and INH.

### 3.3. XRD analyses

The crystallinity behavior of AuNPs/RF/INH/CS-g-PMDA-CYS NP was checked through XRD analysis. The crystalline nature of synthesized AuNPs was observed and shown in Figure S2. AuNPs displayed four different pattern at 2*θ* = 38.1, 44.3, 64.5, and 77.7. All the four pattern related to standard Bragg reflections (111), (200), (220), and (311) of face center cubic (fcc) cross-section (Krishnamurthy et al., 2014). The exceptional diffraction at 38.1 pattern shows that the favored development direction of zero-valent gold was fixed in (111) heading. The amorphous nature of CS-g-PMDA-CYS is shown in Figure S4. In the final XRD, the spectrum shows one broad pattern and the characteristic patterns of AuNPs (Figure S2). This indicates that the crystalline phase of RF and INH may reduce due to successful encapsulation.

**Figure 4.**
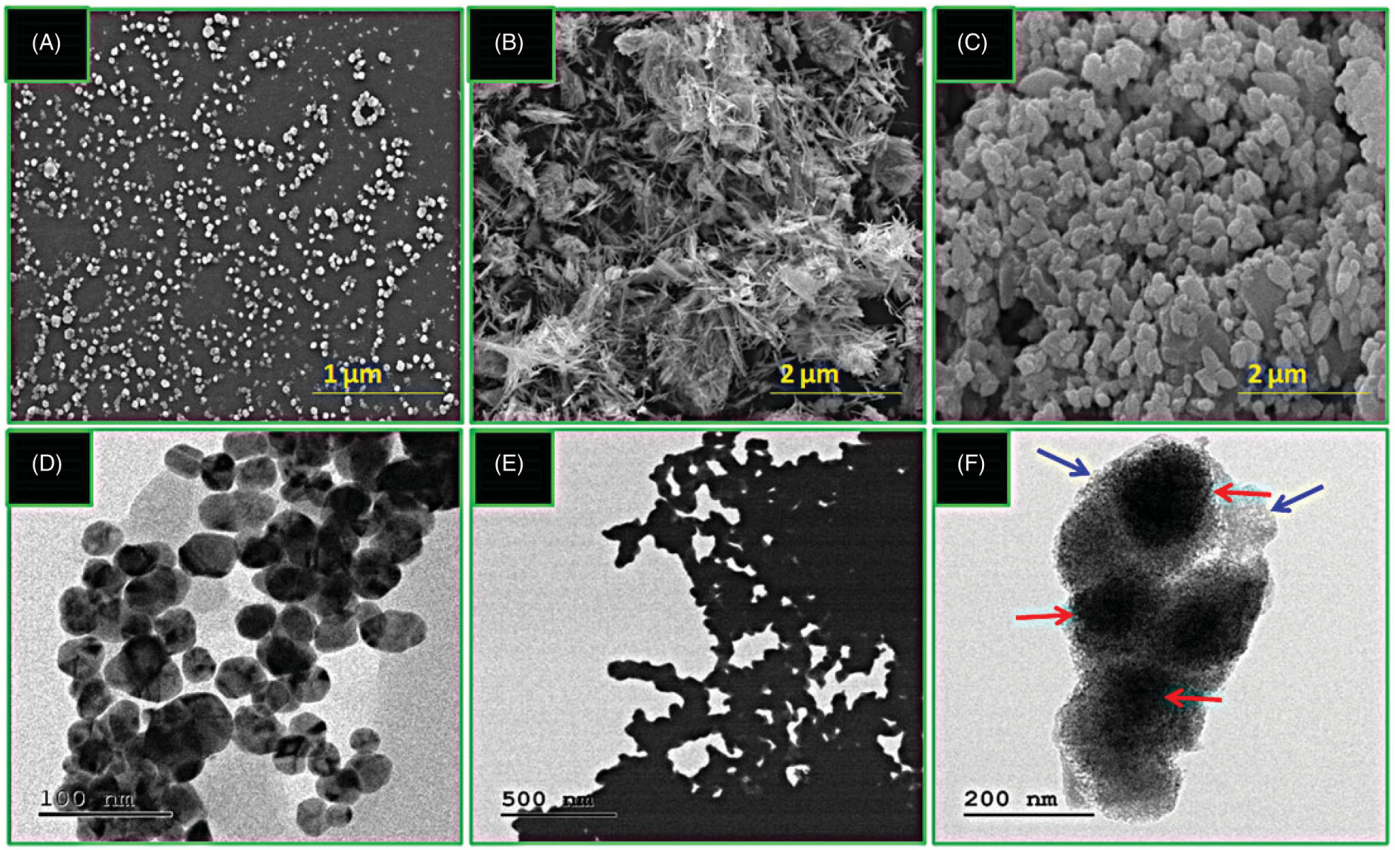
SEM micrographs of (A) Au NPs, (B) CS-g-PMDA-CYS, and (C) AuNPs/RF/INH/CS-g-PMDA-CYS. TEM micrographs of (D) Au NPs, (E) CS-g-PMDA-CYS, and (F) AuNPs/RF/INH/CS-g-PMDA-CYS. The red arrow represents the embedded AuNPs, and the blue arrow indicates the coated CS-g-PMDA-CYS.

### 3.4. SEM, TEM, and AFM analyses

The morphology plays a major character in drug release pharmacokinetics (Sunil et al., 2004). Under SEM observation, AuNPs morphology appears cubical (Figure 4(A)), while CS-g-PMDA-CYS observed a threat like a shape (Figure 4(B)) due to its hydrogel nature. The CS-g-PMDA-CYS conjugated AuNPs moves the morphology toward rough-surfaced nano range (Figure 4(C)). Two factors contribute to this transformation; first, conjugation between AuNPs and –SH functionalized CS-g-PMDA-CYS polymer and second, the capsulation of RF and INH in-between the CS-g-PMDA-CYS and AuNPs. Besides, the morphology observation was further tuned by TEM studies. Cubical shape of AuNP, uniformed thread shape of CS-g-PMDA-CYS polymer, and covering nature of CS-g-PMDA-CYS onto the AuNPs with RF and INH in the morphology of AuNPs/RF/INH/CS-g-PMDA-CYS was confirmed by TEM (Figure 4(D,E,F)). The CS-g-PMDA-CYS covering appears as a cloudy layer onto the AuNPs. The dark core (red arrow) was corresponding to the presence of AuNPs, and the lighter outer core (blue arrow) corresponds to the CS-g-PMDA-CYS.

**Figure 5.**
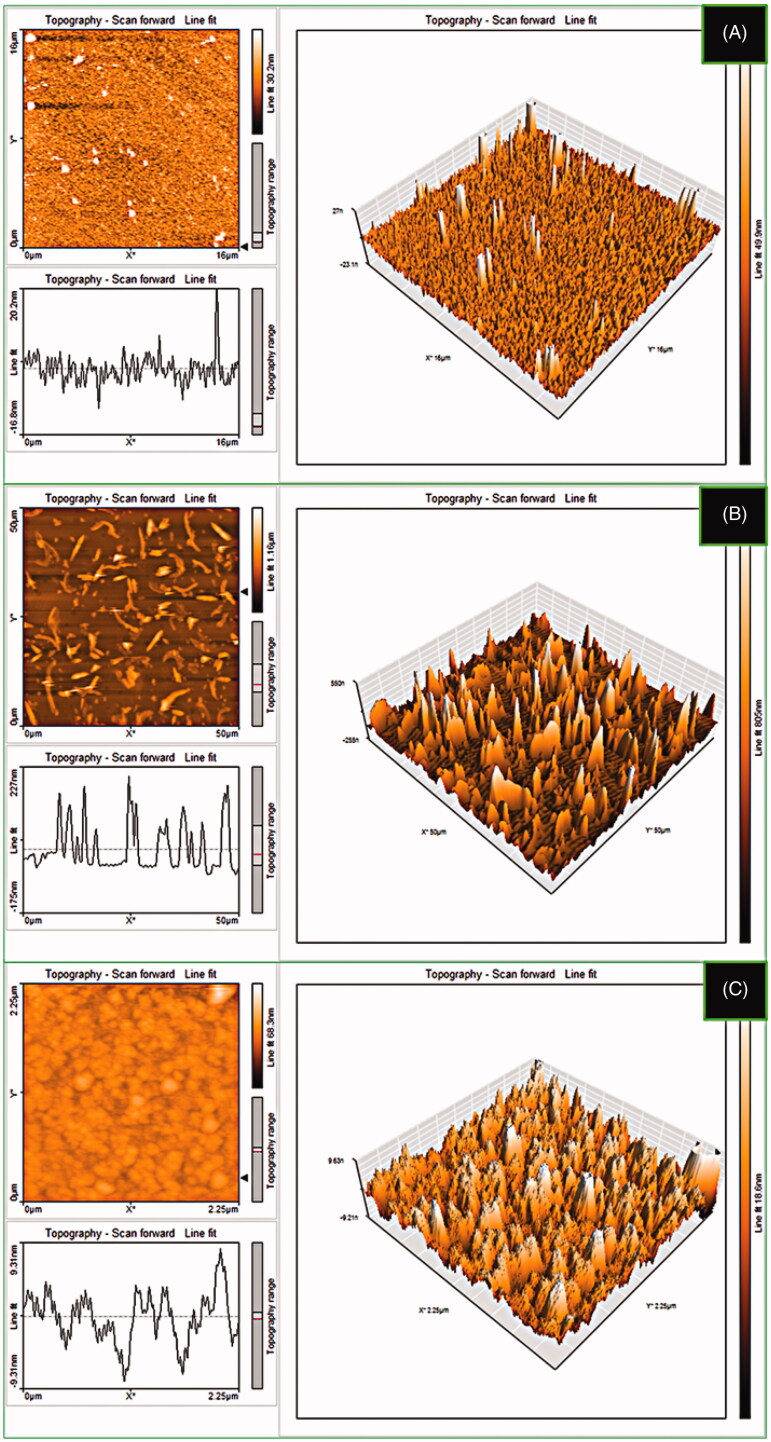
AFM micrographs of (A) Au NPs, (B) CS-g-PMDA-CYS, and (C) AuNPs/RF/INH/CS-g-PMDA-CYS.

The morphology of Au NPs, CS-g-PMDA-CYS, and AuNPs/RF/INH/CS-g-PMDA-CYS was further evaluated by AFM analysis (Figure 5(A–C)). The AFM micrographs of AuNPs were nearly mono-dispersive spherical (Figure 5(A)) while fibril like morphology was obtained for CS-g-PMDA-CYS (Figure 5(B)) and AuNPs/RF/INH/CS-g-PMDA-CYS shows aggregated spherical morphology (Figure 5(C)). Results confirm that the AuNPs/RF/INH/CS-g-PMDA-CYS morphology was aggregated spherical, which can effortlessly enter the cells through the pores.

### 3.5. Particle size and zeta potential

**Figure 6.**
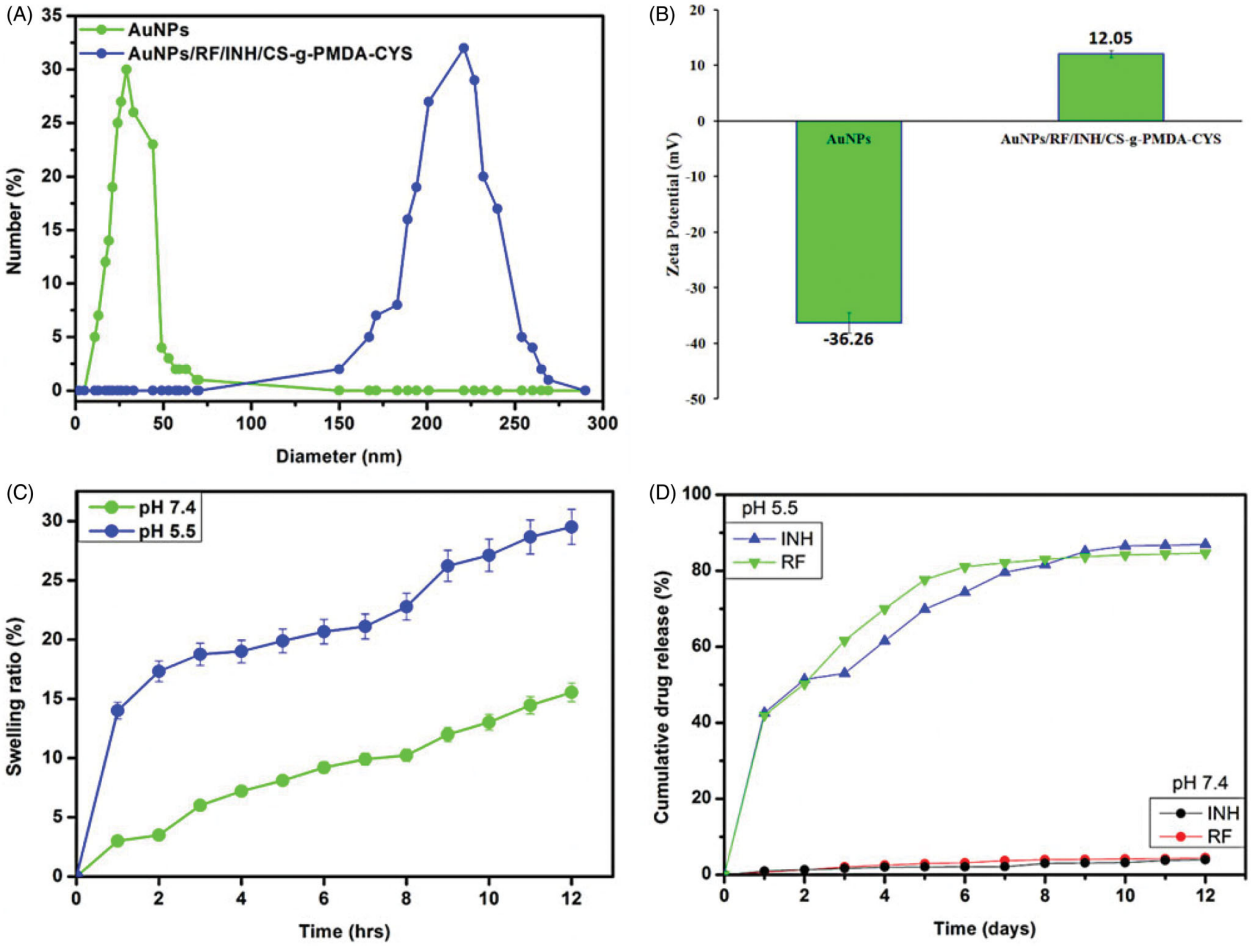
(A) Particle size of AuNPs and AuNPs/RF/INH/CS-g-PMDA-CYS. (B) Zeta potential of AuNPs and AuNPs/RF/INH/CS-g-PMDA-CYS. (C) Swelling profile of AuNPs/RF/INH/CS-g-PMDA-CYS in pHs 5.5 and 7.4. (D) The increasing drug release profile of INH and RF from AuNPs/RF/INH/CS-g-PMDA-CYS at pHs 5.5 and 7.4.

Mean zeta potential and particle size of AuNPs were observed as 26 ± 1 nm and −36.26 ± 0.1 mV, respectively (Figure 6(A,B)). The average particle size for AuNPs/RF/INH/CS-g-PMDA-CYS had increased (231 ± 2 nm), while the zeta potential was decreased (12.05 ± 0.2 mV). The negative zeta potential of AuNPs was suppressed by the conjugation of CS-g-PMDA-CYS and resulted in positive zeta potential.

### 3.6. Swelling analyses

The swelling performance of AuNPs/RF/INH/CS-g-PMDA-CYS was studied in two different pH solutions (Figure 6(C)). The swelling was high due to easy swelling of polymer backbone in acidic medium (pH 5.5) as compared to pH 7.4.

### 3.7. Loading capacity and in vitro drug release pattern

The LC for RF and INH was 9.02% and 13.12%, respectively. The high loading capacity for INH compared with RF was due to the hydrophilic nature of INH. The crucial performance of AuNPs/RF/INH/CS-g-PMDA-CYS in various pH conditions of the human body was systemically analyzed by the *in vitro* method. *In vitro* drug release performance of AuNPs/RF/INH/CS-g-PMDA-CYS was analyzed in two different pH conditions (pHs 7.4 and 5.5) (Figure 6(D)). In the acidic condition (pH 5.5), burst release of RF and INH was observed during initial 24 h followed by a sustained release for 12 days, probably due to pH-responsive nature of –SH, which can easily break in acidic medium and releases RF and INH in a sustained manner. Under human physiological conditions (pH 7.4), the release profile of RF and INH was extremely low (9% and 12%), indicating the stability of AuNPs/RF/INH/CS-g-PMDA-CYS under physiological conditions. These results support that the AuNPs/RF/INH/CS-g-PMDA-CYS is a pH-dependent cargo for the release of drugs.

### 3.8. Anti-pathogenic potential against S. marcescens

#### 3.8.1. Minimum inhibitory concentrations (MIC) determination

The antibacterial and anti-biofilm activity of various nanocomposites has previously been corroborated in several studies. Still, reports on virulence and biofilm inhibitory potential of nanocomposites in contradiction of uropathogens, particularly *S. marcescens* continue naught. Making a prominence for the anti-virulent and anti-biofilm properties of nanocomposites and their practice against infections in modern medications. This work has been made with a key objective to define the effect of AuNPs/RF/INH/CS-g-PMDA-CYS on biofilm and virulence factors of uropathogenic *S. marcescens*. The MIC values achieved for *S. marcescens* were resolved in TSB fluid societies and are expressed in Figure S3. Following 24 h of hatching under oxygen-consuming conditions, variety in the degree of turbidity from clear to shady was seen for all the wells containing nanocomposites demonstrating the development restraint of the microscopic organisms. As appeared, the MIC centralization of AuNPs/RF/INH/CS-g-PMDA-CYS was lower than those obtained for others. When comparing AuNPs/RF/INH/CS-g-PMDA-CYS with INH and RF individually, got an enhanced growing inhibition activity (MIC – 303 μg/ml) for AuNPs/RF/INH/CS-g-PMDA-CYS. Free INH, RF was independently assayed to regulate whether bactericidal natures of AuNPs/RF/INH/CS-g-PMDA-CYS were only originated from nanoparticles.

#### 3.8.2. Inhibition of swarming motility

**Figure 7.**
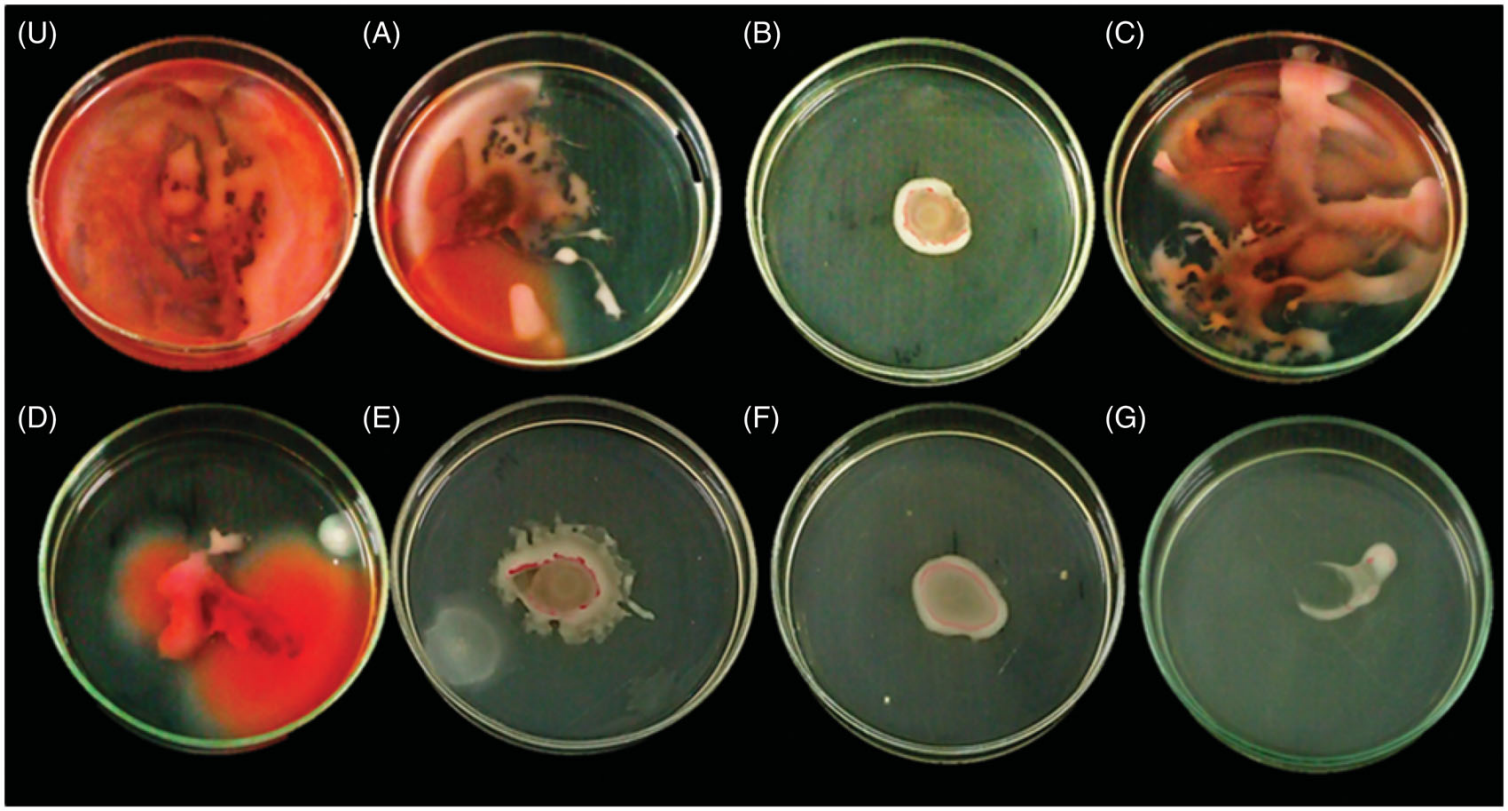
Swarming motility plate of *S. marcescens* Untreated control (U), RF (A), INH (B), AuNPs (C), CS (D), CS-g-PMDA-CYS (E), AuNPs/CS-g-PMDA-CYS, and (F) AuNPs/RF/INH/CS-g-PMDA-CYS (G).

Swarming motility is the typical infectious spectacle of uropathogen, and plays a substantial role in the catheter-associated urinary tract infections. Hence, the competence of *S. marcescens* to swarm over the soft agar swarming plate in the presence of drug combinations was examined (Figure 7). AuNPs/RF/INH/CS-g-PMDA-CYS remarkably repressed the swarming pattern of *S. marcescens* related to control. The presence of AuNPs, RF, INH, and CS-g-PMDA-CYS in the growth medium was originated to constrain the swarming motion of *S. marcescens* typically.

#### 3.8.3. Inhibition of Prodigiosin pigmentation

The pathogenic strains of *S. marcescens* were capable of synthesizing the intracellular pigment Prodigiosin under the direct regulation of the quorum sensing cell to cell signaling machinery. The Prodigiosin quantification assay results exposed that AuNPs/RF/INH/CS-g-PMDA-CYS subdued the prodigiosin pigment synthesis to the level of 76% in the *S. marcescens* compared to other samples (Figure S4).

#### 3.8.4. In situ microscopic observation of biofilm

**Figure 8.**
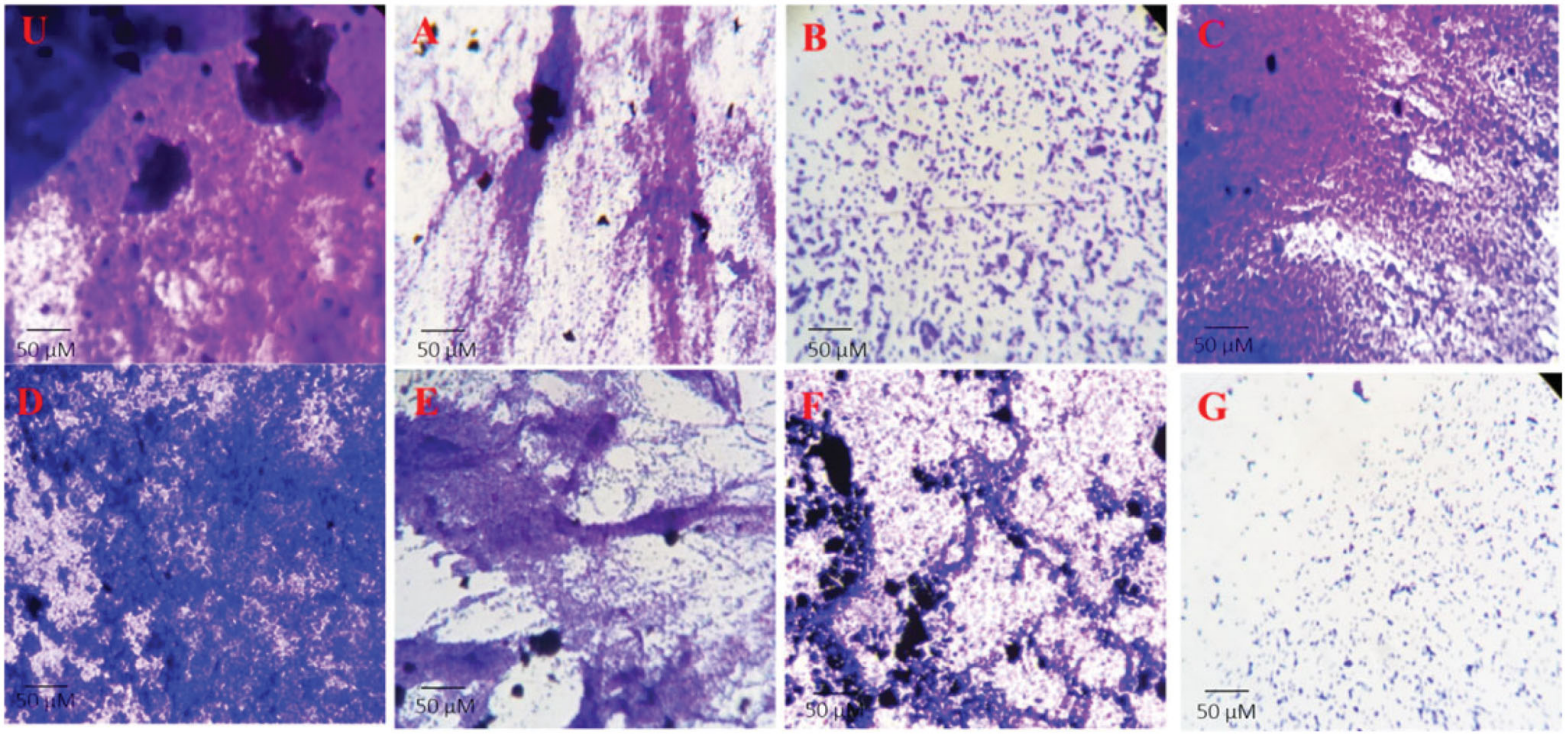
Crystal violet film images (U) RF (A), INH (B), AuNPs (C), CS (D), CS-g-PMDA-CYS (E), AuNPs/CS-g-PMDA-CYS (F), and AuNPs/RF/INH/CS-g-PMDA-CYS (G).

**Figure 9.**
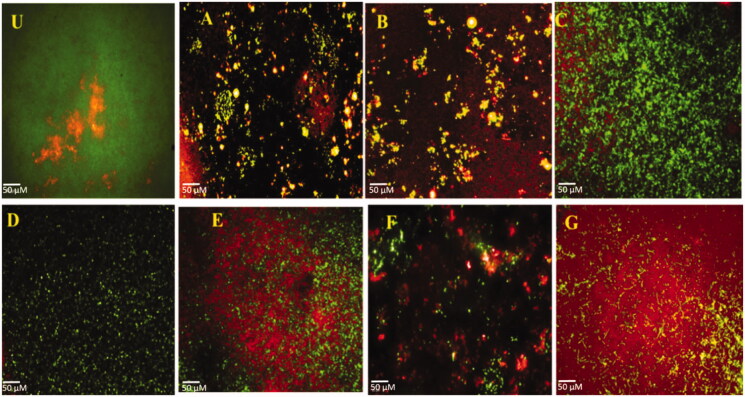
High content screening Live and dead differentiation (U) RF (A), INH (B), AuNPs (C), CS (D), CS-g-PMDA-CYS (E), AuNPs/CS-g-PMDA-CYS (F), and AuNPs/RF/INH/CS-g-PMDA-CYS (G).

The biofilm inhibition potential of RF (A), INH (B), AuNPs (C), CS (D), CS-g-PMDA-CYS (E), AuNPs/CS-g-PMDA-CYS (F), and AuNPs/RF/INH/CS-g-PMDA-CYS (G) at sub MIC levels was established by light, HCS microscopy analysis (Figure 8). The evident decrease was observed in the biofilm construction of the *S. marcescens* in the presence of RF (A), INH (B), AuNPs (C), CS (D), CS-g-PMDA-CYS (E), AuNPs/CS-g-PMDA-CYS (F), and AuNPs/RF/INH/CS-g-PMDA-CYS (G). Upon HCS visualization, the population of dead cells with red fluorescence was significantly elevated after the treatment for all the treatment combinations. Moreover, the construction of the biofilm grieved noticeable modifications, and the volume of dead cells was augmented, especially for AuNPs/RF/INH/CS-g-PMDA-CYS (Figure 9).

### 3.9. Valuation of in vivo therapeutic potential of the drug on C. elegans

#### 3.9.1. Influence of drug on the survival of *C. elegans* upon *S. marcescens* infection

**Figure 10.**
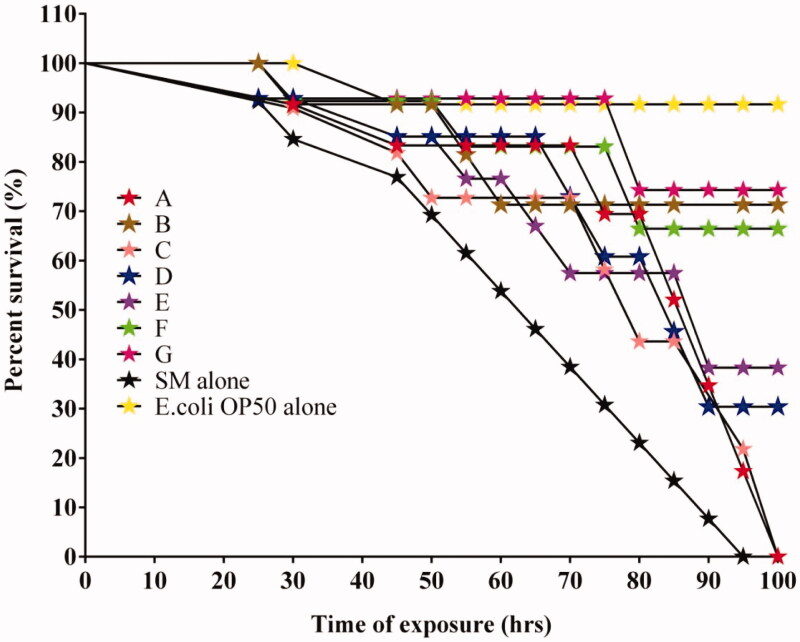
Survival graph presenting the ability of RF (A), INH (B), AuNPs (C), CS (D), CS-g-PMDA-CYS (E), AuNPs/CS-g-PMDA-CYS (F), and AuNPs/RF/INH/CS-g-PMDA-CYS (G) to rescue *C. elegans* from *S. marcescens* infection.

*Caenorhabditis elegans* is a perspective in *vivo* therapeutic model organism routinely practiced to assess the pharmacological and toxicity properties of drug-loaded samples. The exponential improvement of nanotechnology requires the utilization of option *in vivo* model frameworks to evaluate the toxicity impacts of the integrated nanomaterial. The consequences of challenge endurance measure uncovered that *S. marcescens* is increasingly pathogenic to *C. elegans* since comprehensive death was detected at 93 h in *S. marcescens* infected *C. elegans* groups (Figure 10). Remarkably, AuNPs/RF/INH/CS-g-PMDA-CYS escalates 74% of *C. elegans* survival during *S. marcescens* infection.

#### 3.9.2. Microscopic observation of in vivo colonization

**Figure 11.**
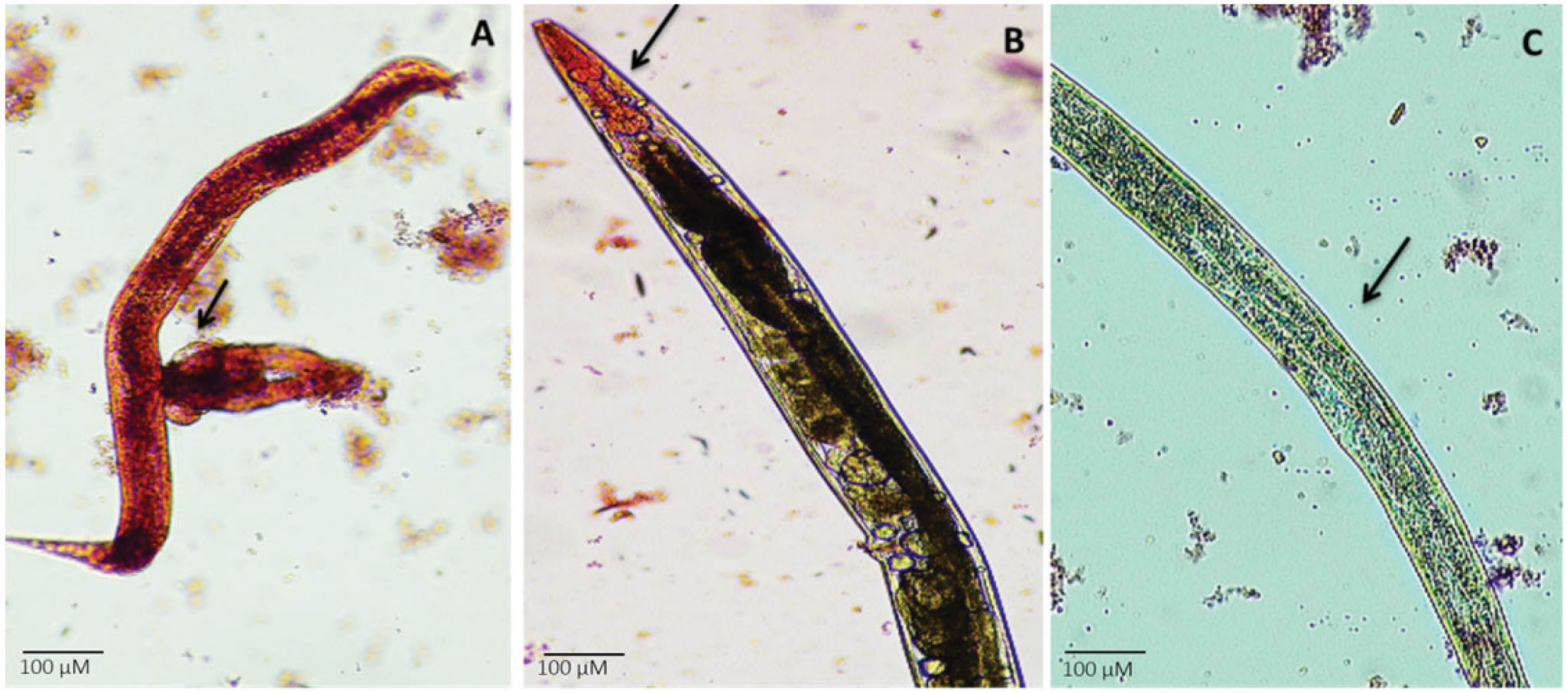
Light micrographs portray the reticence of Prodigiosin pigment (B and C) and intestinal colonization of *S. marcescens* upon AuNPs/RF/INH/CS-g-PMDA-CYS treatment when compared to control (A).

Light microscopy images revealed that the presence of AuNPs/RF/INH/CS-g-PMDA-CYS negatively influences the production of prodigiosin, which eventually leads to the condensed intestinal colonization in *C. elegans*. Clear pink coloration and colonization were witnessed in the whole body and intestinal area of *C. elegans* during *S. marcescens* infection (Figure 11(A) – control). However, no such coloring was observed in *C. elegans* with the existence of AuNPs/RF/INH/CS-g-PMDA-CYS and *S. marcescens* together. Besides, *C. elegans* infected with *S. marcescens* showed injured pharynx and intestine (Figure 11(A)). On the other hand, *C. elegans* fed with AuNPs/RF/INH/CS-g-PMDA-CYS + *S. marcescens* showed characteristic pharynx and well-organized body (Figure 11(B,C)). Hence, it is clear that AuNPs/RF/INH/CS-g-PMDA-CYS raises the existence of *C. elegans* by directing biofilm development and virulence in *S. marcescens.*

## 4. Conclusion

Here, we report the formulation, characterization, and biological evaluation of multi drugs combined with AuNPs-fused polymeric drug delivery system. The chemical modifications, drug interactions, crystallinity, and morphology of the synthesized drug carrier systems were evaluated with a series of physiochemical characterizations. Higher drug release from AuNPs/RF/INH/CS-g-PMDA-CYS was obtained in an acidic environment. The AuNPs/RF/INH/CS-g-PMDA-CYS showed good bactericidal/antimicrobial activity in both gram-positive (*Staphylococcus aureus*), and Gram-negative (*K. pneumoniae)* and the results implement the studies of AuNPs/RF/INH/CS-g-PMDA-CYS for further biological analysis. The *in vivo* performance on *S. marcescens* indicates the localization effect of AuNPs/RF/INH/CS-g-PMDA-CYS, and it supersedes drug resistance of *S. marcescens* by inhibiting swarming motility and subdued prodigiosin pigment synthesis. The current study can open-up a meadow for the development of an effective drug delivery system in drug-resistant microbial management. The authors suggest further studies for a better understanding of the cytotoxicity of the proposed drug delivery model.

## Associated content

### Supporting information

Encapsulation, *In vitro* drug release profile, FT-IR and XRD results.

## Supplementary Material

Supplemental Material
