# Supplementary material for: A promising drug delivery candidate (CS-g-PMDA-CYS-fused gold nanoparticles) for inhibition of multidrug-resistant uropathogenic Serratia marcescens
Source: Drug Deliv. 2020 Sep 4;27(1):1271–82. doi: 10.1080/10717544.2020.1809557 (PMC8216475; doi:10.1080/10717544.2020.1809557)
Supplement: Supplemental Material [file IDRD_A_1809557_SM9540.docx]

**A promising Drug Delivery candidate (CS-g-PMDA-CYS fused Gold Nanoparticles) for inhibition of multi drug resistant uropathogen (*Serratia marcescens)***

Ping Shi^a^, Rajendran Amarnath Praphakar^b^, Sadhasivan Deepa^b^, Kannan Suganya^c^, Prashant Gupta^d^, Riaz Ullah^e^, Ahmed bari^f^, Marudhamuthu Murugan^c^, Mariappan Rajan^a^*

^a^ Management Office of Drug Clinical Trial Research, Affiliated Hospital of Qingdao University, 266001, Qingdao, Shandong

^b^***** Biomaterials in Medicinal Chemistry Laboratory, Department of Natural Products Chemistry, School of Chemistry, Madurai Kamaraj University, Madurai-625021, India.

^c^ Department of Microbial Technology, School of Biological Sciences, Madurai Kamaraj University, Madurai, Tamil Nadu 625021, India.

^d^ Department of Balroga, Govt. Ayurved College, Raipur, Chhattisgarh, India

^e^ Department of Pharmacognosy, College of Pharmacy, King Saud University Riyadh, Saudi Arabia

^f^ Department of Pharmaceutical Chemistry,  College of Pharmacy, King Saud University Riyadh, Saudi Arabia.

Corresponding Author

^*^Biomaterials in Medicinal Chemistry Laboratory, Department of Natural Products Chemistry, School of Chemistry, Madurai Kamaraj University, Madurai-625021, India.

E-mail: rajanm153@gmail.com; Tel: +91 9488014084, Fax: 0452–2459845

***

***

**Figure S1.** The preparation of AuNPs from HAuCl_4_.





**Figure S2.** FT-IR spectrum of RF and INH.





**Figure S3.** XRD spectrum of RF and INH.


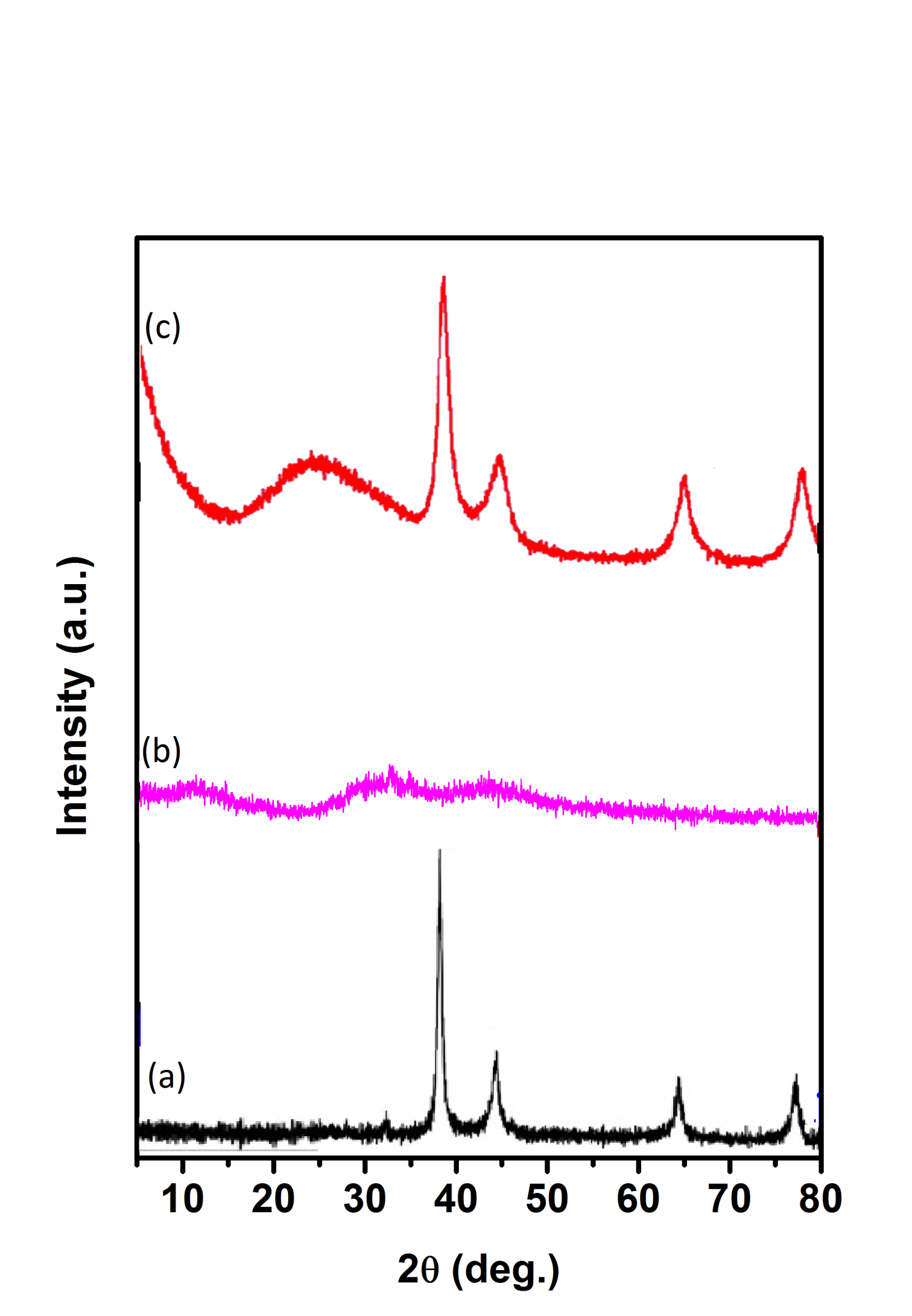


**Figure S4.** XRD images of (a) AuNPs, (b) CS-g-PMDA-CYS and (c) AuNPs/RF/INH/CS-g-PMDA-CYS NPs.


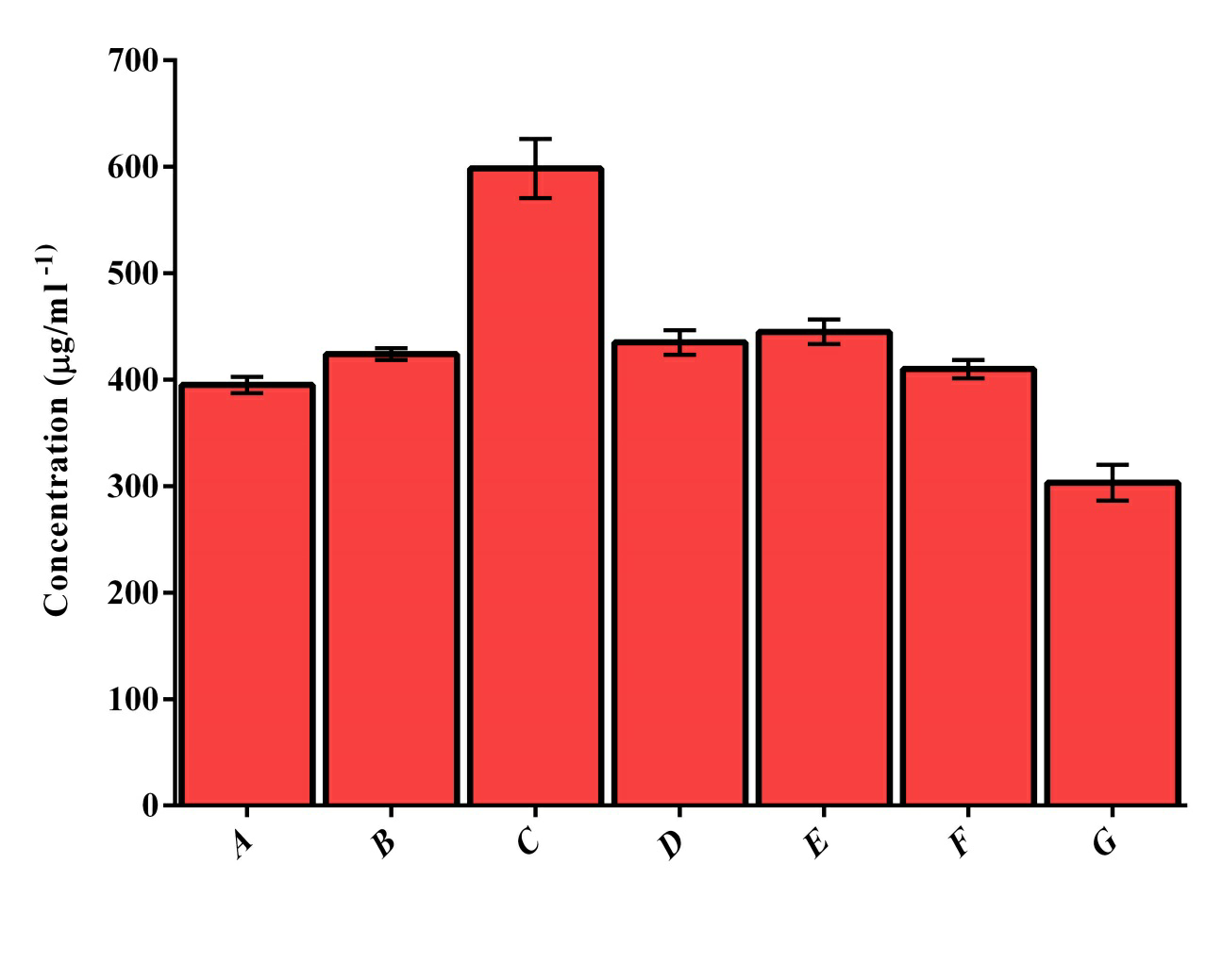


**Figure S5.** MIC of RF (A), INH (B), AuNPs (C), CS (D), CS-g-PMDA-CYS (E), AuNPs/CS-g-PMDA-CYS (F) AuNPs/RF/INH/CS-g-PMDA-CYS (G) against *Serratia marcescens*


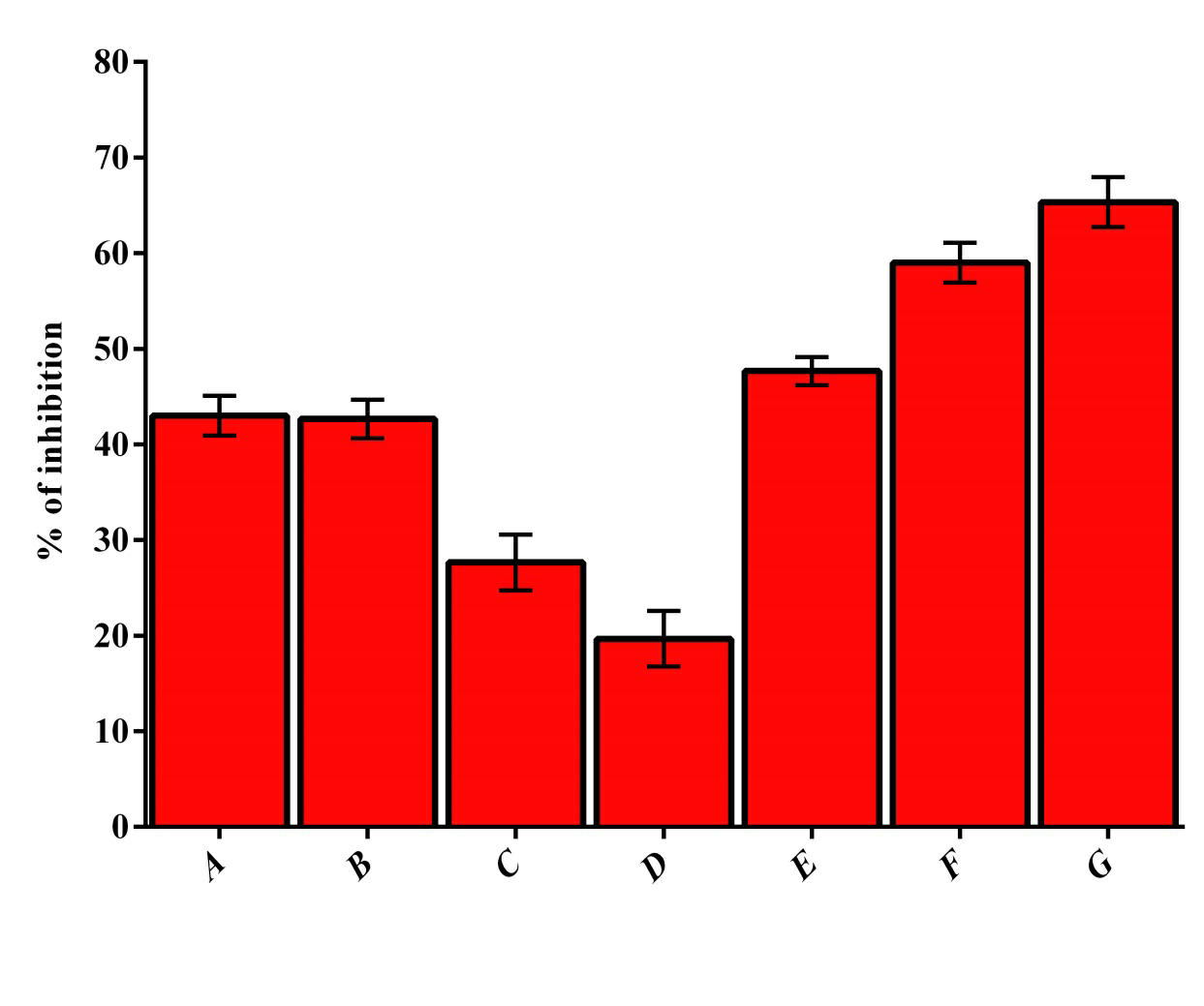


**Figure S6.** Inhibition of Prodigiocin rate RF (A), INH (B), AuNPs (C), CS (D), CS-g-PMDA-CYS (E), AuNPs/CS-g-PMDA-CYS (F) AuNPs/RF/INH/CS-g-PMDA-CYS (G).
